# Perceptions and Attitudes towards Mules in a Group of Soldiers

**DOI:** 10.3390/ani11041009

**Published:** 2021-04-03

**Authors:** Javiera Lagos, Manuel Rojas, Joao B. Rodrigues, Tamara Tadich

**Affiliations:** 1 Programa Doctorado en Ciencias Silvoagropecuarias y Veterinarias, Universidad de Chile, Santiago 8820808, Chile; jalagos@ug.uchile.cl; 2 Departamento Ingeniería Industrial, Facultad Ciencias Físicas y Matemáticas, Universidad de Chile, Santiago 8320198, Chile; manuelrojas@uchile.cl; 3 Research and Operational Support Department, The Donkey Sanctuary, Sidmouth, Devon EX10 0NU, UK; joao.rodrigues@thedonkeysanctuary.org.uk; 4 Instituto de Ciencia Animal, Facultad de Ciencias Veterinarias, Universidad Austral de Chile, Valdivia 5090000, Chile

**Keywords:** mule, animal welfare, empathy, soldiers, human–animal interactions, perceptions

## Abstract

**Simple Summary:**

Working equids play an essential role in the livelihoods of millions of families around the world. The way people, especially their caretakers, perceive them affects attitudes towards them and consequently their welfare. This study aimed to understand the perceptions and attitudes of soldiers towards the mules they work with. For this, psychological constructs, such as empathy and pain perception, and discourse analysis were used. The results show that soldiers’ empathy towards animals is positively associated with their perception of pain and empathy towards humans. Soldiers prefer to work with mules over donkeys and horses, and perceive mules as intelligent and with the best aptitudes for pack work in the mountains, although they perceive them as aggressive. The text analysis shows that soldiers have a good understanding of mules’ nutritional, environmental and health needs but require a better understanding of their behavioral and emotional needs. Finally, they see mules as strong and noble animals, valuable to work under difficult field conditions and an essential component that supports army logistics in the mountain. Future selection and training strategies for soldiers should include behavior and welfare concepts to facilitate the soldier–mule relationship and improve mules’ welfare.

**Abstract:**

Mules are essential for pack work in mountainous areas, but there is a lack of research on this species. This study intends to assess the perceptions, attitudes, empathy and pain perception of soldiers about mules, to understand the type of human–mule relationship. For this, a survey was applied with closed-ended questions where the empathy and pain perception tools were included and later analyzed through correlations. Open-ended questions were analyzed through text mining. A total of 73 soldiers were surveyed. They had a wide range of ages and years of experience working with equids. Significant positive correlations were found between human empathy, animal empathy and pain perception. Soldiers show a preference for working with mules over donkeys and horses. Text mining analysis shows three clusters associated with the mules’ nutritional, environmental and health needs. In the same line, relevant relations were found for the word “attention” with “load”, “food”, and “harness”. When asked what mules signify for them, two clusters were found, associated with mules’ working capacity and their role in the army. Relevant relations were found between the terms “mountain”, “support”, and “logistics”, and also between “intelligent” and “noble”. To secure mules’ behavioral and emotional needs, future training strategies should include behavior and welfare concepts.

## 1. Introduction

Equids have participated in the development of practically every aspect of human civilization [1]. There are an estimated 200 million equids worldwide (horses, mules and donkeys) mainly distributed in middle and low-income countries. These animals play a major role in different work types [2], either by load-pulling or pack work. The direct or indirect incomes generated by these activities support the subsistence of approximately 600 million people [3].

Working equids are considered as an essential component of livelihood and can be considered part of the natural, physical, social and financial capitals of people, increasing the resilience capacity of communities [4,5]. The social and economic contribution of equids to livelihoods can be direct (provision of transportation services) or indirect (plowing the land for obtaining agricultural products) [6]; thus, underestimating their impact could negatively affect society [7]. The main welfare problems reported in working equids include lesions due to poor harnessing, dehydration, hoof and shoeing problems, poor body condition scores and behavioral problems, such as aggressiveness [5,8,9]. Although the amount of studies reporting the effectiveness of training strategies, including control groups, is scarce, Stringer et al. [10] showed that a significant change in the knowledge of owners could be achieved by different types of interventions. Similarly, Degefa et al. [11] showed that the training of farriers resulted in improvements in animal-based welfare indicators.

Equids also remain being used as key elements for human military purposes [12], playing a major role as animals of burden, messengers and protectors [13]. They also assume a key role as means of transport during natural disasters, when the army needs to access remote areas to rescue people or provide supplies. Mules, in particular, have been essential in transporting equipment, soldiers and their supplies [14]. This role assumes a redoubled importance in countries, such as Chile, with a long mountainous chain that can be of difficult access for mechanized vehicles and even for horses.

Mules have been considered to be superior to horses for pack work due to their higher endurance capacity, the better quality of hooves, fewer feed requirements, and higher longevity/working life span [15,16]. In mountainous areas, they have a more secure step and a temperament that makes them an excellent pack animal compared to horses [16]. Studies in different countries have shown a predilection for mules over donkeys and horses, but mules are usually less available and thus considered more valuable [17]. On the other hand, they are usually considered more aggressive than horses, making them a difficult animal to work with [18].

The welfare of equids, in this case, mules, can be affected by how they interact with their caregiver, the knowledge the person has on husbandry practices and behavior, and also on how motivated the person is about working with an animal; therefore, positive human–animal interactions can potentially increase productivity [8,19,20,21]. How a person perceives animals can be influenced by knowledge and by the person’s affective states; this is important since perception can influence decision-making in relation to husbandry practices. Thus, recognition of pain and the capacity to be affected by and share the emotional state of another [22] allows taking decisions, such as providing rest, water, pain relief, among others. Perception of animals can also be influenced by previous experiences, culture, religion, and knowledge; hence perception can move from a totally instrumental view of the animal up to a more affective view. Training strategies should be designed considering these perceptions since it increases the likelihood of their success [23].

Text analysis performed using text mining strategies appears as a powerful tool that can allow extracting information from open-ended questions. For example, latent semantic analysis extracts and infers relationships of expected contextual usage of words in passages of discourse [24]. Word and passage meaning representations that result from applying LSA are capable of simulating diverse human cognitive phenomena, including discourse comprehension, analysis of interviews and free text surveys, quantitative literature reviews, and analysis of textual data, among others [24,25]. In spite of its potential, LSA has received little attention; in the field of animal welfare and human–animal relationship, it has been used to analyze how working horses’ owners perceive their animals [26].

The human–animal relationship is based on multiple events, allowing animals to memorize and, more importantly, predict future interactions, thus having important implications for animal welfare [27]. In the case of the army mules, handlers or caregivers constantly change in time, not necessarily allowing the formation of the human–animal relationship, and such fact could have negative effects on the mules’ welfare state. Soldiers working with mules are not responsible for ensuring the acquisition of resources for the mules since the army provides all resources. Moreover, they do not receive specific training in animal welfare aspects, such as mules’ behavioral needs and basic handling of them. The aim of the present study is to assess the perceptions, attitudes, empathy and pain perception of soldiers about the mules they work with. This will allow a better understanding of the type of human–mule relationship that they form while working together.

## 2. Materials and Methods

This study was approved by the Institutional Committee for the Care and Use of Animals and Bioethics (No.19263-VET-UCH).

A questionnaire was constructed using the Google Forms tool to assess attitudes, perceptions, empathy and pain recognition of a group of soldiers. The questionnaire consisted of six sections, with closed-ended and open-ended questions. The first section included an informed consent of soldiers. After its approval, demographic information was consulted, such as age, sex and level of training within the army. The second section included closed-ended questions on their attitudes towards mules, perceptions about mules and species preference (donkeys, horses or mules) at the moment of working with equids. The third section corresponded to the human–animal empathy scale [28], and the fourth to the brief form of the Davis interpersonal reactivity index instrument (B-IRI) [29,30]. The fifth section was on the perception of the equine pain scale (PEPS). The last section included two open-ended questions (a) “In your opinion, which are the most important resources that mules need to be able to work?” and (b) “What do mules represent or mean for you?” The questionnaire was shared with the soldiers associated with functions with equids, and 73 responses were obtained between March and April of 2020.

### 2.1. Assessment of Militaries Human–Animal Empathy

A modified version of the human–animal (H–A) empathy scale developed by Paul [28] was used. The instrument was previously modified for its use in Spanish and applied to persons related to equids and with low-level education [21], considering that these instruments need to be designed according to the population in which they are applied [31]. The H–A empathy scale applied consisted of eleven statements concerning animals, all of them suggesting empathic feelings. Response to each statement was in the form of a 9 points Likert scale, where 1 corresponded to “very strongly disagree” and 9 to “very strongly agree”. The final scores of the H–A empathy scale were calculated as the sum of the eleven responses of each respondent. Scores on the empathy scale can range from 11 to 99, with higher scores indicating higher levels of human–animal empathy [21].

### 2.2. Assessment of Militaries Human–Human Empathy

To determine the level of human–human (H–H) empathy, the brief form of the Davis interpersonal reactivity index (B-IRI) [29,30] was applied. The Spanish version of this instrument has been validated in Chile for university students [32] and already applied to horse owners in Chile by Luna et al. [21]. The B-IRI consists of 16 items that had to be scored on a five-point Likert scale, where 1 was “does not describe me well” and 5 “describes me well”. Scores on the B-IRI can range from 16 to 80, with higher scores indicating higher levels of human–human empathy.

### 2.3. Assessment of the Perception of the Intensity of Pain in Equids by Soldiers

A previously constructed instrument perception of equine pain scale (PEPS) was applied [21,33]. The scale consisted of 17 color photographs that showed equids suffering from a range of different conditions implying varying intensities of pain, including management procedures, infectious diseases and traumatic injuries. Soldiers were instructed to rate the photographs according to their perception of pain intensity with a five-point Likert scale, where 1 was “no pain” and 5 “maximum possible pain”. The final score for each respondent was calculated as the sum of the 17 responses. Scores for the PEPS can range from 17 to 85, with higher scores indicating a greater pain perception.

### 2.4. Data Handling for Closed-Ended Questions

Data were analyzed with descriptive statistics and then Spearman’s correlations were calculated due to its nonparametric distribution. The strength of the relationship between variables (very low = 0.00 to 0.19; low = 0.20 to 0.39; modest = 0.40 to 0.69; high = 0.70 to 0.89; very high = 0.90 to 1.00) was defined based on the Spearman rank correlation coefficient, according to Cohen and Holliday [34].

### 2.5. Text Mining of the Open-Ended Questions

Text mining includes a series of techniques that allow the assessment of nonstructured information, mainly texts. Text mining allows the discovery of previously unknown information within the text and transforms it into data for analysis. In this study, the following techniques were applied: preprocessing of data; term frequency; clustering; semantic correlations and latent semantic analysis. The Kamada–Kawai method [35] and the Calinski method [36] were applied for processing the cluster analysis.

In the determination and implementation of the different semantic algorithms of this study, the open-source software (RStudio 1.2.5, Ide development) was used, and the packages are detailed in Table 1.

To predict the answer to the closed-ended question, “Do you like to work with mules?” (yes or no), the answer to each of the open-ended questions was analyzed independently through traditional techniques of machine learning. In this case, the random forest technique was applied. Then the results were analyzed using a confusion matrix (CM).

## 3. Results

A total of 73 soldiers responded to the questionnaire; from these, 98.6% (*n* = 72) were men, there was only one woman. Results are presented in sections according to the types of questions: closed-ended and open-ended questions.

### 3.1. Closed-Ended Questions

The median age of respondents was 36 years (27–54 years), and the experience with equids varied according to the species; most soldiers had experience with horses, followed by mules and then donkeys, but the range of years varied from no experience at all (0 years) to up to 30 years of experience (Table 2).

From the total respondents, 89% (*n* = 65) said they did like to work with mules, while 11% (*n* = 8) said they did not like to work with them. Among soldiers, who did not like working with mules, the frequency of interaction ranged from weekly (two individuals) to once a year. In terms of training, 98.6% (*n* = 72) had received some type of training with equids at the army, and 69.8% (*n* = 51) had done some type of training with equids as civilians.

When scoring the photos according to the level of pain perceived (Table 3), conditions, such as open fracture of the tibia, articular capsule wound, laminitis with hoof growth and saddle sore, presented the lowest variance and the highest scores of perceived pain. On the other hand, hoof overgrowth, dermatophilosis, poor trimming and shoeing, and pectoral burn wound presented the highest variability, with pain scores ranging between 1 and 5.

A significant but modest correlation was found between the pain perception of the soldiers and their human–animal empathy (*p* < 0.001) and also a significant but modest correlation between human–animal and human–human empathy (r = 0.408; *p* < 0.001) (Table 4).

The number of training courses in different aspects of equid management varied from 0 training to 6 courses. The amount of training did not correlate to any of the variables, except with experience with mules, where those soldiers with higher experience with mules had a higher number of courses, although this correlation was low (r = 0.29, *p* = 0.012). The PEPS scores and the empathy scores (both H–H and H–A) were compared between respondents that had veterinary nurse training and those that did not. No significant differences were found for PEPS (*p* = 0.487), H–H empathy (*p* = 0.425) nor H–A empathy (*p* = 0.183).

Mules were the preferred species to work with by the respondents; soldiers also believed that they had a better aptitude for working in the mountains, for pack work and better physical resistance. Although they considered mules the most aggressive equid among the three options, they also considered them the most intelligent ones when compared to horses and donkeys. Donkeys were the least preferred by the soldiers, except aggressiveness, where they obtained the same percentage as horses (Table 5).

### 3.2. Open-Ended Questions

**Figure 1:**
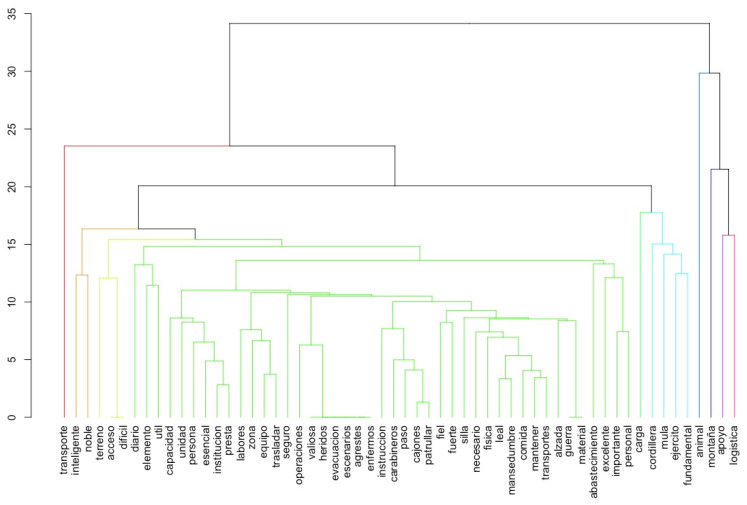
Dendrogram of words for the question, “What do mules represent or mean for you?” The y-axis represents the Euclidean distance (dissimilarity level), and the x-axis the word clusters. Words are in Spanish to not alter the dendrogram.

For the question, “In your opinion, What are the most important resources that mules need to be able to work?”, the recognition of the mule’s basic needs by the soldiers was analyzed. For the frequency of terms analysis, food, water and supplements were the most frequent terms included in the soldier’s answers. The term food was the most relevant, followed by a relationship between water and forage and a relationship between alfalfa and concentrates. These relationships were visible when constructing the words’ dendrogram (Figure 1). The cluster analysis resulted in 9 clusters, from which the first three are presented in Table 6, with the three main words included in each. The optimal number of clusters was obtained through the Calinski shortest dendrite method.

The correlation analysis for this question shows a high correlation between “attention” and “paddocks” (r = 1), “attention” and “food troughs” (r = 1); “mule” and “personnel” (r = 0.9) “shoeing” and “harness” (r = 0.81), and between “deteriorated” and “harness” (r = 0.81).

The latent semantic analysis provides latent relationships, and not direct ones, within a text based on the construction of particular vectorial spaces. The asymmetry metric measures semantic similarity between words with a proximity in distributional space and identifies hypernyms [37]. The asymmetry metric for words of interest is shown in Table 7; the results show a more relevant relationship between “attention–load” (0.483), “attention–harness” (0.071), “attention–shoeing” (0.071) “food–water” (0.492), “food–supplement” (0.363) and “food–cleanliness” (0.343).

For the question, “What do mules represent or mean for you?” the most frequent words were “animal”, “mountain”, and “support”. The dendrogram analysis (Figure 1) for these questions reveals the relevance of the term animal (in Spanish “animal”) and a relationship between the terms mountain, support and logistics (in Spanish “montaña”, “apoyo”, and “logística”). In a similar way, the terms transport, intelligent and noble (in Spanish, “transporte”, “inteligente”, and “noble”) are closely related. Applying the Kamada–Kawai method [35], the semantic network also showed a close relationship between animal, useful and support (in Spanish, “animal”, “útil” and “apoyo”). Using the Calinski criteria [36] for obtaining clusters, two clusters could be observed (Table 8).

When applying the correlation analysis, a very high correlation was observed between “field” and “scenario”, “sick”, “evacuation”, “injured” and “valuable” (r = 1), a high correlation between “transport” and “operations” (r = 0.78), and between “path” and “Andes” (r = 0.72). A modest correlation was found between “unit” and “mule” (r = 0.62) and between “mule” and “Andes” (r = 0.58). When using latent semantic analysis, the asymmetric similarities shown in Table 9 were observed. The most relevant relations were between “animal–noble”, “animal–intelligent” and “animal–load”, while “animal–friend” obtained the lowest closeness and relevance index (0.212). The relevance of each word’s meaning is characterized by its loading on each of the dimensions in the latent semantic space.

The random forest technique, a traditional machine learning technique, was applied to perform a prediction process using the answers to open-questions 1 and 2. In this case questions, 1 and 2 were used to predict the response to the closed-ended question “Do you like to work with mules?”. For question 1, “In your opinion, what are the most important resources that mules need to be able to work?”. The model could predict with an exactitude of 86.7% positive answers but 0% for negative answers. For question 2, “What do mules represent or mean for you?” it was also able to predict the positive answers 80% of the time but could not predict negative answers.

## 4. Discussion

Mules are the least studied equids [38], but they have gained increasing attention in the scientific literature in the last decade in terms of their physiology and behavior. The aim of adding knowledge about the mule-human interaction is that the present study analyzed attitudes and perceptions of a group of soldiers towards mules.

Pain perception, human–human and human–animal empathy of the participants were assessed since these human psychological attributes can modulate the human–animal interaction and thus affect animal welfare [39]. The human–animal empathy scores obtained in this study were lower than those reported by Luna et al. [21] for working horse owners in Chile. These horses are usually considered as part of the family by their owners [26], and a closer contact can allow owners to better understand their horse’s needs and identify them. Mules have been described as routine makers and forming strong bonds with their caretakers, especially when they are handled from young ages [18,40], something not observed in the army. Temporal and handling routines generate a predictable environment for the animal [41]. Unpredictable environments have been described as a single acute stressor that disrupts normal activities, with negative welfare implications [42]. In the army, mules are randomly assigned to soldiers that do not necessarily use the same signaling systems when handling them or that can change the temporality of husbandry practices, such as feeding, reducing predictability and therefore, hindering the formation of the human–animal bond. This could explain the lower human–animal empathy scores found in the soldiers compared to working horse owners.

In terms of pain perception, the soldiers scored on average higher scores than working horse owners and equine practitioners from Chile [21,33]. Hackamore wounds and dermatophilosis were assigned the lowest scores with a median score of three out of five, conditions, which were also perceived as low pain conditions in horses by equine practitioners [33]. On the other hand, the highest pain scores with the lowest variance were assigned to the photographs of an open fracture of the tibia, scored as maximum possible pain by all soldiers and a wound with exposure of the articular capsule (mean score of five with a range between four and five. Pain assessment is difficult and requires an observer very acquainted with the species [43,44]. The lack of in-depth training in health-related issues, welfare and behavior, could affect the pain scoring provided by the soldiers. Not understanding the physiological processes behind these conditions could signify under or over-estimating welfare issues or not providing rapid treatment when required. Hemsworth and Coleman [45] indicate that stockpersons should be able to quickly identify any changes in the behavior, health or performance of the animals to seek support. In this study, the soldiers tended to overestimate the pain in some of the assessed conditions, thus increasing the probability of veterinary treatment, although this relationship needs to be evaluated in further studies. In spite of the greater pain perception, there is still a great variability for some conditions, similar to what was observed in other studies [21,44,46]. These findings could reflect a lack of experience in diseases of low incidence or insufficient training in health-related issues, welfare and behavior. As in Luna et al. [21] and Ellingsen et al. [44], significant correlations were found for human–animal empathy level and pain perception, indicating that individuals with higher levels of human–animal empathy could be more sensitive to animal pain perception. Moreover, human–animal empathy had a significant, positive, and moderate association with human–human empathy, indicating a relationship between both concepts, similar to previous studies [21,28,47]. Empathy is affected by multiple factors like gender, experience, education and animal species [47,48]; thus, someone’s empathy level cannot be associated with one single factor. For example, it has been indicated that the typical driver of a brick kiln donkey is an adolescent with very little experience in how to fairly handle an equid and with very little motivation in securing animal welfare [8]. By including empathy in the strategies applied to train these drivers, one could improve their motivation and their attitudes towards their equids, thus improving welfare. In studies with urban working horses (cart horses), it was determined that neither the socioeconomic status nor the quality of life of owners could predict the welfare state of the horses [5]. On the other hand, it has been described that owners with higher levels of human–animal empathy and higher perception of pain kept working horses with a better animal welfare index. Moreover, human–animal empathy explained 60% of the animal welfare state [21].

The majority of soldiers perceived mules as more aggressive than horses and donkeys and with the lowest market value. Nevertheless, when it comes to aptitudes related directly to their work, soldiers considered mules better for pack work, with higher physical resistance and the most intelligent ones among the three species, therefore, preferring to work with them. These results align with the literature where mules have been claimed to be the most important animal the army has ever had, with an important role in peacetime as pack animals [49]. The army transport mule has been described as temperamental, unfriendly and difficult to work with [49]. Army training manuals from India and Britain emphasized that mules must be treated gently if an effective human–animal working partnership was the aim and that rough treatment had been proved fatal for successful training [50].

This somehow portraits the special temperament of mules. Historically there has been a cultural bias against mules and in favor of horses provided in different contexts. For example, in the colonial times in America, the hierarchy between the Spanish elite, Mulato laborers and the indigenous workforce was rendered as an analogy of the hierarchy between horses, mules and donkeys [51]. In the same line, recent studies show that handlers frequently describe mules as difficult to handle and aggressive [18,38,40]. Contrary, in the present study, a negative bias was found for donkeys, and horses were only perceived as more expensive and less aggressive than mules.

The discourse analysis applied shows that the soldiers surveyed understand the needs of mules, especially regarding the nutritional, environmental and health-related domains. The cluster analysis identified three clusters from the answers given to the open-ended question associated with, “What are the most important resources that mules need to be able to work?”. Cluster 1 can be associated with the environmental domain, cluster 2 to the nutritional domain and the last one to the health domain according to the five domains model first proposed in 1994 [52] and recently revised in 2020 [52,53]. The same can be appreciated in the latent semantic analysis where the words of interest are “attention” and “food”, with the first one being associated with objective words associated with the environment and health aspects (load, harness, shoeing, health), while the food was associated with objective nutritional words (water and supplement). Coincidently, Pinsky et al. [54] assessed attitudes towards health and husbandry of working ponies in Indonesia, and their results showed that working horses’ drivers identified vitamin injections and appropriate diet as the most important needs, without considering behavioral or emotional needs.

The behavioral and emotional needs of mules were not represented by the soldier’s answers, somehow pointing to the lack of training in applied behavior and welfare aspects during the soldier’s formation process. Mellor et al. [53] highlight the importance of the behavioral interactions of animals, either directed to other animals, including humans or the environment, since when they can achieve their behavioral goals, then positive emotions can be enhanced and thus their welfare. Rodrigues et al. [9] also highlighted the pivotal role of understanding human–animal interactions and human behavior change in enhancing the welfare of working equids.

The answers to the questions “What are the most important resources that mules need to be able to work?” and, “What do mules represent or mean for you?” were able to predict in over 80% of the times a positive answer to the question “Do you like to work with mules?”, but were not able to predict negative answers. This could be due to the low frequency of negative answers in the studied group but should be further studied since it could allow a better selection of soldiers that will then be assigned to work with equids. Working with animals places different demands on people, and there is a need to identify the best attributes to meet the job requirements [55]. The soldiers will not only be exposed to adverse climate and geographical conditions, heavy loads, including uniforms and supplies that need to be transported, but also they need to understand the mules’ needs. Hemsworth and Coleman [45] propose several characteristics for a stockperson that would probably also apply to soldiers, such as general knowledge of the nutritional, climatic, social and health requirements of the animal; practical experience in the care of the animal; ability to quickly identify any changes in the behavior and health of the animals, and the ability to work independently and as a team for the care and maintenance of the animals. If some or all of these attributes could be recognized during the selection process, it would then facilitate the required training process in specific tasks associated with the animals and potentially benefit the mules’ welfare.

Finally, when asking the soldiers, “What do mules represent or mean for you?” the most frequent terms were associated with their work role in the mountains as pack animals that provide support and ease logistics. This can be seen through the very high correlation between words, such as field, scenario, sick, injured evacuation or between transport and operations. The soldiers seem to have a very instrumental view of the mules they work with. Similar results are shown through the cluster analysis, the dendrogram and the latent semantic analysis where the words animal–friend obtained the lowest closeness and relevance index. This is in line with the description of army mules provided through history, where they have been described as “the most important four-legged animal the army had ever had” or as a “splendid” means of transport and “source of general admiration” [49]. The words used by the soldiers to describe the mules they work with differ from those used by urban working horses asked the same question, where horses are viewed as a friend, family member and important income generation tool [26]. One important difference between the present study and previous ones studying perceptions towards working or pleasure/sport equids [21,56] is that, in the case of the army mules, the caretakers are not the owners and do not receive direct income from the mules. Such fact could be the main difference in the perception and attitudes towards them, resulting in a more instrumental and utilitarian view.

The present study is the first approach to understand the perceptions that soldiers have about mules. Nevertheless, it must be acknowledged that it has limitations, such as the sample size. Latent semantic analysis has been mostly used as a methodology for quantitative literature reviews and analysis of textual data. Although analysis of interviews is an area of application, more methodological development work is still necessary for this direction, being this an approximation to its potential use.

## 5. Conclusions

The present study shows that the soldiers have high levels of human–animal empathy, which is significantly correlated with their capacity to perceive pain in equids. Although they perceive mules as more aggressive than horses and donkeys, they have a higher preference for working with them, considering them intelligent and with better aptitudes for work. This could represent a more instrumental view of mules and explain that they can recognize mules’ basic needs associated with adequate nutrition, health and environment (housing), but there is a lack of understanding about their behavioral and emotional needs. Future selection and training strategies for soldiers should include behavior and welfare concepts since terms associated with these aspects did not appear in their answers. These are essential for allowing better human–animal interaction during work, avoiding accidents for both soldiers and mules and improving the welfare of the mules. This becomes even more important for mules’ case since they have been described as routine makers able to form strong bonds with their caretakers.

## Figures and Tables

**Table 1:** Open-source software used for text mining. The library, version and its objective are shown.

Library	Version	Objective
Cluster	2.1.0	Cluster package
Corrplot	0.84	Correlation matrix package
Tm	0.7–6	Text mining package
Ggplot2	3.2.1	Graphics package
Igraph	1.2.4.1	Graphics package
Tcltk	3.5.1	Interface package
Lsa	0.73.1	Lsa package
LSAfun	0.6	Lsafun package
RStudio	1.2.5	Ide development

**Table 2:** Age and years of experience of soldiers working with horses, mules or donkeys (*n* = 73). Levels of empathy and pain perception are also described here

	Mean (SD)	Median	Min	Max
Age (years)	36.6 (5.9)	36	27	54
Years of Experience				
Horses	13.4 (9.8)	14	0	30
Mules	11.5 (8.4)	11	0	30
Donkeys	4.6 (8.6)	0	0	30
Empathy				
H–A (11–99)	75.8 (13.5)	77	35	97
H–H (16–80)	51.8 (9.2)	52	30	80
Pain Perception				
PEPS (17–85)	73.8 (7.4)	74	51	85

H–H = human–human empathy scale; H–A = human–animal empathy scale; PEPS = perception of the equine pain scale. Means, standard deviation (SD), median, minimum and maximum are included.

**Table 3:** Distribution of the soldiers (*n* = 73) according to pain score (1 = “no pain; 5 = maximum possible pain) assigned to each of the 17 photographs of painful conditions presented to them. Variance, median and range are provided.

	Likert-Type Scale							
Painful Conditions	1	2	3	4	5	Variance	Median	Range
Open fracture of the tibia	0	0	0	0	73	0	5	5–5
Articular capsule wound	0	0	0	8	65	0.09	5	4–5
Laminitis with hoof loss	0	0	1	3	69	0.16	5	3–5
Saddle sore	0	0	2	5	66	0.17	5	3–5
Septic arthritis	0	0	3	4	66	0.20	5	3–5
Subsolar abscess	0	0	4	12	57	0.31	5	3–5
Mastitis	0	1	4	16	52	0.43	5	2–5
Fetlock rope burn	0	0	7	13	53	0.43	5	3–5
Evisceration	1	1	4	6	61	0.57	5	1–5
Cannon and pastern rope burn wound	0	1	14	20	38	0.69	5	2–5
Saddle sore	1	1	14	27	30	0.77	4	1–5
Castration	1	1	12	20	39	0.79	5	1–5
Hackamore burn wound	1	13	31	12	16	1.13	3	1–5
Pectoral burn wound	1	7	14	18	33	1.17	4	1–5
Poor trimming and shoeing	3	4	13	17	36	1.27	4	1–5
Dermatophilosis	21	12	29	7	4	1.36	3	1–5
Hoof overgrowth	2	10	13	19	29	1.37	4	1–5

**Table 4:** Spearman’s correlation matrix (R-values) for the pain perception, human–human (H–H) and human–animal (H–A) empathy tools, age and experience with horses, mules and donkeys.

	Experience Horses	PEPS	Age	H–A Empathy	H–H Empathy	Exp. Mules
Pain perception (PEPS)	−0.1751					
Age	0.3616 **	0.0431				
H–A empathy	0.2058	0.4054 **	0.0782			
H–H empathy	0.0561	−0.0294	0.1689	0.4080 **		
Experience w/mules	0.4364 **	0.0012	0.7291 **	0.0746	0.1134	
Experience w/donkeys	0.3382 **	0.0518	0.3170 **	0.0687	0.1672	0.2860 *

* *p* < 0.05; ** *p* < 0.001.

**Table 5:** Soldiers species preference according to each statement. Total frequency and percentage are provided.

From the Following Species, Which:	Horses	Mules	Donkeys	All of Them
You prefer to work with…	29 (39.73%)	37 (50.68%)	0 (0%)	7 (9.59%)
Has better aptitude for pack work…	2 (2.74%)	71 (97.26%)	0 (0%)	0 (0%)
Has better resistance for work…	5 (6.85%)	67 (91.78%)	0 (0%)	1 (1.37%)
Has the best market price…	72 (98.63%)	1 (1.37%)	0 (0%)	0 (0%)
Has better aptitudes for work in mountains…	0 (0%)	72 (98.63%)	0 (0%)	1 (1.37%)
You consider the most aggressive…	5 (6.85%)	58 (79.45%)	5 (6.85%)	5 (6.85%)
You consider the most intelligent…	27 (39.99%)	40 (54.79%)	2 (2.74%)	4 (5.48%)

**Table 6:** Description of the three clusters and the principal words that describe the cluster.

Clusters	Three Principal Words in the Cluster
Cluster 1	adequate, attention, enclosure
Cluster 2	water, food, adequate
Cluster 3	food, care, shoeing

**Table 7:** Results of the latent semantic analysis in terms of word of interest and objective word and their respective asymmetry metric for the question, “What are the most important resources that mules need to be able to work?”

Word of Interest	Objective Word	Asymmetry Metric
attention	load	0.483
attention	harness	0.071
attention	shoeing	0.071
attention	hoof trimming	0.063
attention	health	0.037
attention	grooming	0.022
food	water	0.492
food	supplement	0.363
food	cleanliness	0.343
food	quality	0.326

**Table 8:** Description of the two clusters and the principal words that describe the cluster.

Clusters	Three Principal Words in the Cluster
Cluster 1	animal, support, capacity
Cluster 2	animal, army, support

**Table 9:** Results of the latent semantic analysis in terms of word of interest and objective word and their respective asymmetry metric for the question What do mules represent or mean for you?

Word of Interest	Objective Word	Asymmetry Metric
animal	noble	0.692
animal	intelligent	0.672
animal	load	0.661
animal	mountain	0.620
animal	army	0.601
animal	support	0.535
animal	equipment	0.437
animal	loyal	0.421
animal	friend	0.212

## Data Availability

The data presented in this study are available on request from the corresponding author. The data are not publicly available due to confidentiality agreement included in the informed consent.
